# X-ray driven peanut trait estimation: computer vision aided agri-system transformation

**DOI:** 10.1186/s13007-022-00909-8

**Published:** 2022-06-06

**Authors:** Martha Domhoefer, Debarati Chakraborty, Eva Hufnagel, Joelle Claußen, Norbert Wörlein, Marijn Voorhaar, Krithika Anbazhagan, Sunita Choudhary, Janila Pasupuleti, Rekha Baddam, Jana Kholova, Stefan Gerth

**Affiliations:** 1 Development Center for X-Ray Technology (Entwicklungszentrum Röntgentechnik, EZRT), Fraunhofer Institute for Integrated Circuits (Institut Für Integrierte Schaltungen, IIS), Flugplatzstraße 75, 90768 Fürth, Germany; 2 Crops Physiology & Modeling, Accelerated Crop Improvement Research Theme, International Crops Research Institute for the Semi-Arid Tropics (ICRISAT), Hyderabad, 502324 Telangana India; 3 Universität Osnabrück, 49069 Osnabrück, Germany; 4 Department of Information Technologies, Faculty of Economics and Management, Czech University of Life Sciences Prague, Kamýcká 129, Prague, 165 00 Czech Republic

**Keywords:** Peanut production, Technology-driven system transformation, X-ray, Convolutional neural network (CNN), Kernel weight, Shelling percentage

## Abstract

**Background:**

In India, raw peanuts are obtained by aggregators from smallholder farms in the form of whole pods and the price is based on a manual estimation of basic peanut pod and kernel characteristics. These methods of raw produce evaluation are slow and can result in procurement irregularities. The procurement delays combined with the lack of storage facilities lead to fungal contaminations and pose a serious threat to food safety in many regions. To address this gap, we investigated whether X-ray technology could be used for the rapid assessment of the key peanut qualities that are important for price estimation.

**Results:**

We generated 1752 individual peanut pod 2D X-ray projections using a computed tomography (CT) system (CTportable160.90). Out of these projections we predicted the kernel weight and shell weight, which are important indicators of the produce price. Two methods for the feature prediction were tested: (i) X-ray image transformation (XRT) and (ii) a trained convolutional neural network (CNN). The prediction power of these methods was tested against the gravimetric measurements of kernel weight and shell weight in diverse peanut pod varieties^1^. Both methods predicted the kernel mass with R^2^ > 0.93 (XRT: R^2^ = 0.93 and mean error estimate (MAE) = 0.17, CNN: R^2^ = 0.95 and MAE = 0.14). While the shell weight was predicted more accurately by CNN (R^2^ = 0.91, MAE = 0.09) compared to XRT (R^2^ = 0.78; MAE = 0.08).

**Conclusion:**

Our study demonstrated that the X-ray based system is a relevant technology option for the estimation of key peanut produce indicators (Figure 1). The obtained results justify further research to adapt the existing X-ray system for the rapid, accurate and objective peanut procurement process. Fast and accurate estimates of produce value are a necessary pre-requisite to avoid post-harvest losses due to fungal contamination and, at the same time, allow the fair payment to farmers. Additionally, the same technology could also assist crop improvement programs in selecting and developing peanut cultivars with enhanced economic value in a high-throughput manner by skipping the shelling of the pods completely.

This study demonstrated the technical feasibility of the approach and is a first step to realize a technology-driven peanut production system transformation of the future.

**Supplementary Information:**

The online version contains supplementary material available at 10.1186/s13007-022-00909-8.

## Introduction/background

Markets and value-chains linked to agricultural produce face many irregularities related to misrepresentation of the raw produce value [1, 2]. Such irregularities are common during procurement and, whether intentional or not, are percolating the emerging markets resulting in tremendous financial losses for the individual companies [3–8]. A key factor causing procurement irregularities is the non-transparent estimation of the commodity price. When coupled with poor value-chain logistics and a lack of storage facilities, which is common for the emerging markets, further challenges related to commodity quality and safety arise [3, 9–11]. Unfortunately, within the current agricultural commodity trade the primary producers are affected the most.

Several technologies have been used to mitigate hurdles in the agricultural commodities trade related to standardization assessments of commodity values and/or safety [12–15]. However, these technologies include classical destructive manual or laboratory testing methods (e.g., gravimetry, DNA sequencing, mass spectroscopy, biochemical analyses) but also consider the indirect methods for commodity evaluation based on sensors (e.g., near infra-red or X-ray spectroscopy) [3, 12–14, 16]. The technology driven solutions, especially the portable ones, are in the spotlight of the international authorities as they might provide effective means to fill the blank spots of various agricultural value-chains [12, 16–19]. Within these, the X-ray-based systems are being used for non-destructive inspections of food matter structure, density, composition and homogeneity [20–28] and are used for many applications related to standard grain evaluation and inspection [29–42]. Although X-ray systems are traditionally stationary, the recent technology advancement highlighted the technology can be mobilized for a range of out-doors applications. The CTportable series is one of the examples [43] demonstrating that it is possible to scale the system in terms of size and throughput for dedicated use-cases. With these kinds of X-ray systems, it is possible to skip the destructive part of material evaluation—i.e., the shelling of peanuts in this example. One of the major points is always the radiation protection needed to assure a safe operation with these mobile systems. However, not only portable CT devices but also handheld X-ray fluorescence scanners are already available. Nevertheless, the mere availability of these kinds of systems does not imply the easy and fast detection of price indicators. For this, adapted imaging pipelines are needed to generate the relevant price indicator out of the captured raw data.

**Fig. 1:**
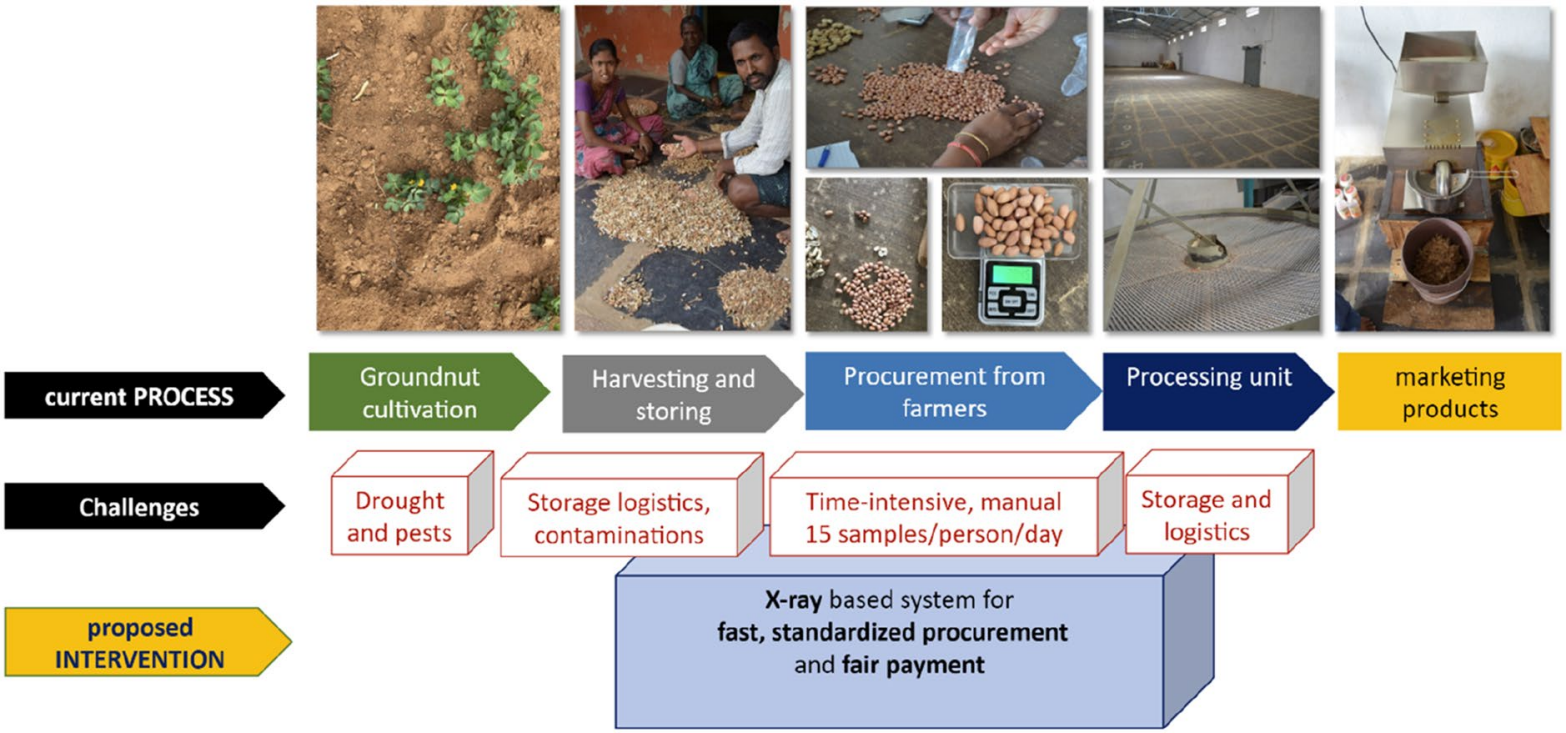
Graphical abstract showing the differences of the current process of evaluating produce compared to the described technology-based process as presented in this publication

In the case study presented, we investigated whether an X-ray-based system is a suitable technology option to assess the peanut commodity price indicators (Fig. 1). As a demonstration use-case, we selected the peanut value-chain in the Kalyandurg *mandal* (14.55°N, 77.11°E, 656 m; an administrative division of Anantapur district, Andhra Pradesh, India). In this case, the commodity is procured by aggregators from the farmers in their fields in the form of whole peanut pods. The procurement costs depend mostly on subjective visual evaluation (physical contaminations, damages, kernel size) and shelling percentage (kernel weight/total pod weight estimated gravimetrically) estimated by the aggregator [44, 45]. The pooled produce is then transported to a processing unit where the raw peanut is shelled, kernels mechanically graded and further sold based on the features of recovered kernels (physical properties and biochemical composition). At present, the procurement speed does not guarantee that all farmers in the region can be visited on time. This can result in crop value deteriorations—mainly fungal contamination [46–49]. We also argue that substituting the manual procurement method with suitable technology could, in the future, standardize and accelerate the procurement process and, at the same time, allow fair-procurement cost estimation for the producers while avoiding the produce deterioration.

Herewith, we present a proof-of-concept study which investigates whether the 2D X-ray scans of whole peanut pods in combination with several feature prediction algorithms can be used to predict peanut attributes that are important for peanut commodity price estimates (i.e., kernel weight and shell weight).

## Results

### Peanut varieties evaluation for price indicators: kernel weight, shell weight, shelling percentage

**Fig. 2:**
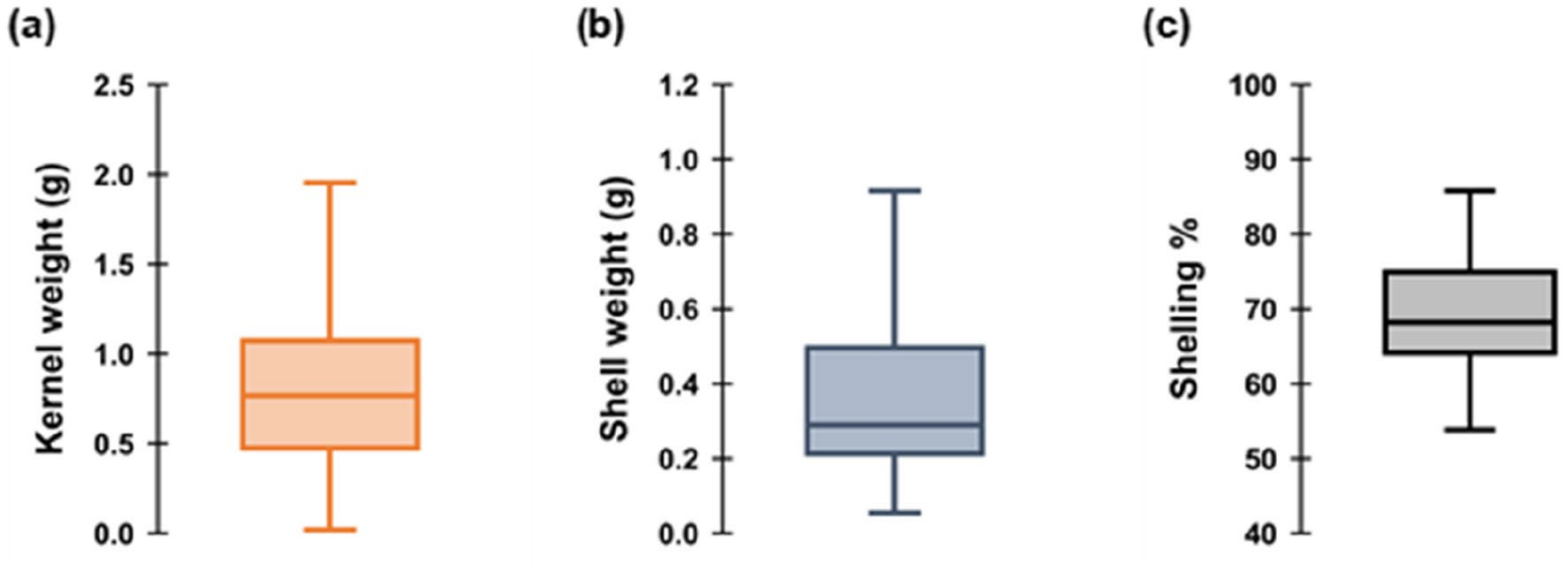
Boxplots depicting the distribution and range of kernel weight (**a**), shell weight (**b**), and shelling percentage (**c**) across 39 diverse peanut varieties assessed in the study. Kernel and shell weight were quantified by gravimetric measurements as a ground truth and shelling percentage was calculated as the ratio of kernel weight to total pod weight. Details on the varieties used are further elaborated in Additional file 1: Table 1 and Fig. S1 a–c

Three market price driving features of peanut pods; i.e., kernel weight (g/pod), shell weight (g/pod) and shelling percentage (100*total kernel weight/total pod weight) were analyzed in the study (details in "Peanut commodity price indicators"). The evaluated kernel weights ranged from 0.003 g to 2.47 g/pod with the average of 0.83 g/pod (Fig. 2a, Additional file 1: Table S1). Similarly, the minimum shell weight in the studied dataset was 0.05 g/pod while the maximum was 1.40 g/pod with an average of 0.38 g/pod (Fig. 2b, Additional file 1: Table S1). Shelling percentage—another parameter of economic importance—spanned across 1.5% to the maximum 87.8% with an average of 67.65% (Fig. 2c, Additional file 1: Table 1). The distributions of values for all three features in the analyzed dataset were skewed towards lower values (i.e., the lower values were over-represented in the dataset, Fig. 2a–c) which had significant implications for the construction of the CNN feature predicting algorithms (i.e., the importance of each value for CNN construction was weighted based on the frequency of its abundance in the dataset; see "Peanut pod features prediction from X-ray scans through image transformation (XRT) and a convolutional neural network (CNN) regression model", "Feature predictions methods and comparison metrics"). The in-depth analysis further revealed there were significant differences among the 39 investigated varieties in all three characters: kernel weight, shell weight and shelling percentage (Additional file 1: Fig. S1a–c).

### Peanut pod features prediction from X-ray scans through image transformation (XRT) and a convolutional neural network (CNN) regression model

**Fig. 3:**
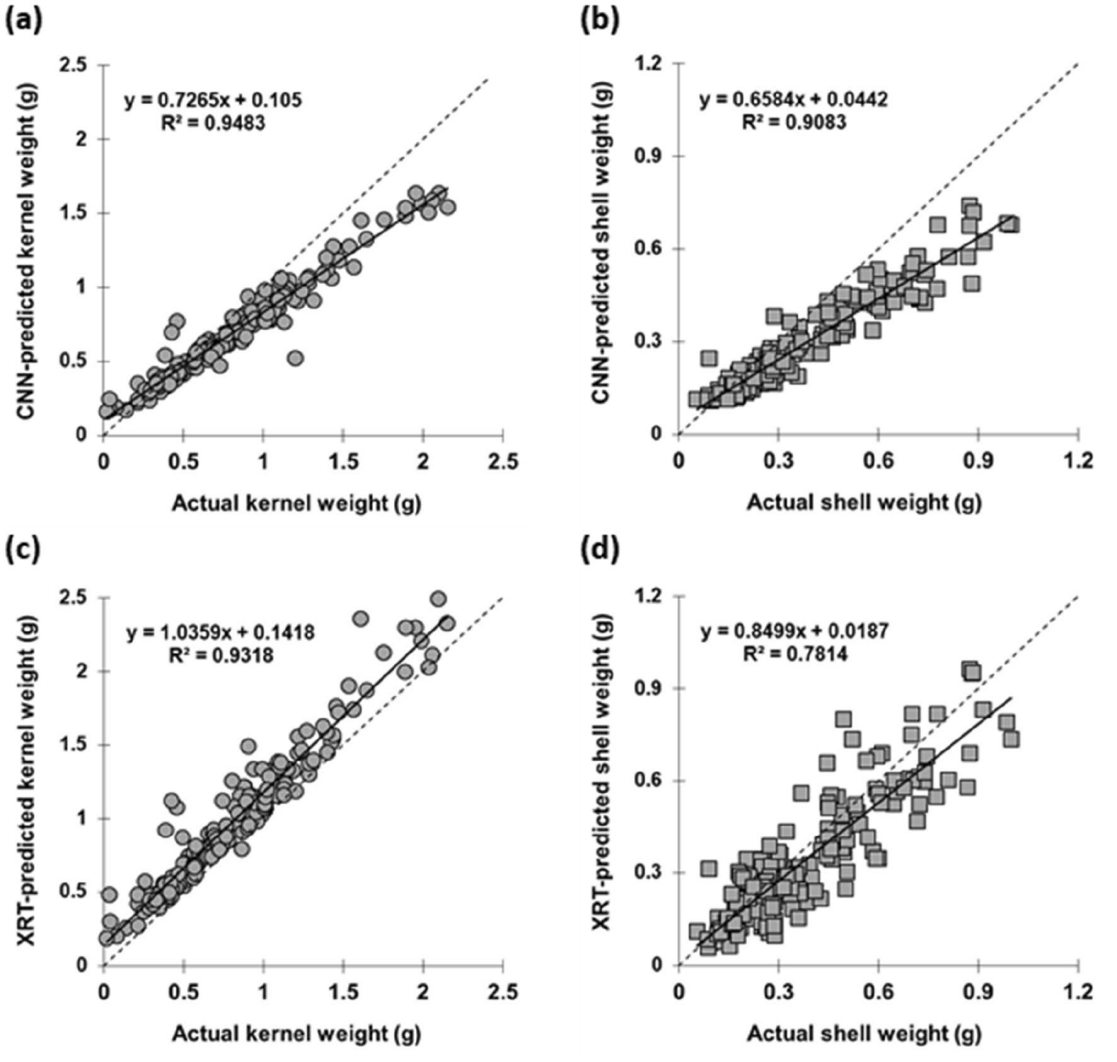
Regression plots illustrating the prediction power of CNN **a**, **b** and direct X-ray images transformation (XRT; **c**, **d**) models for kernel weight (**a**, **c**, solid circle) and shell weight (**b**, **d**, solid square) parameters. Graphs visualize the relation of the 10% of the dataset (“test set”) to the actual gravimetrically estimated ground truth which was used to infer the statistical metrics. The expanded statistical metrics of this dataset is summarized in Table 1b. Kernel weight (solid circle); Shell weight (solid square); Solid line (—) depicts the linear regression between the ground truth observations and predictions; Dashed line (---) indicates the 1:1 relation

The peanut pods were scanned using the X-ray system CTportable160.90 ("2D X-ray images" section). The resulting X-ray projections were pre-processed ("Image treatment" section) and, consequently, two methods have been used to predict the peanut pod characters: kernel weight and shell weight (X-ray image transformation: "Features prediction using X-ray transformation (XRT) of images", and convolutional neural network: "Features prediction using CNN regression model"). To express prediction accuracies and enable the comparison of the two pod feature prediction methods, the descriptive statistics of linear correlation function between values predicted by X-ray image transformation (XRT) and the Convolutional Neural Network (CNN) method with ground truth measurements were used (Table 1a,b, Fig. 3, details in "Feature predictions methods and comparison metrics" section).

**Table 1:** a,b Summary of statistical indicators used to evaluate the prediction power of the direct X-ray images transformation (XRT) model and the CNN model for inferring the kernel weight and shell weight from 2D X-ray scans

	Kernel weight/pod (by XRT method)	Kernel weight/pod (by CNN method)	Shell weight/pod (by XRT method)	Shell weight/pod (by CNN method)
(a) Metrics: calibration				
r	0.93	0.97	0.84	0.94
R^2^	0.87	0.95	0.71	0.89
MSE	0.06	0.03	0.02	0.02
MAE	0.18	0.14	0.09	0.1
Slope	1.01	0.75	0.78	0.64
Intercept	0.16	0.09	0.04	0.05
(b) Metrics: testing				
r	0.97	0.97	0.91	0.96
R^2^	0.94	0.94	0.82	0.92
MSE	0.05	0.07	0.01	0.03
MAE	0.18	0.21	0.08	0.1
Slope	1.03	0.73	0.84	0.61
Intercept	0.15	0.1	0.02	0.05

These are: r (Pearson’s correlation coefficient), R^2^ (coefficient of determination), MSE (mean squared error), MAE (mean absolute error), slope and intercept of relation between the ground-truth observations (kernel and shell weight) and predictions (by XRT and CNN model). The metrics specific to calibrations set (90% of dataset) is in Metrics: calibration, while the metrics of the test set (testing set) is in Metrics: testing

This included splitting the data set into training (see Table 1a) and test (see Table 1b, Fig. 3) set which included 90% and 10% of the data set, respectively., For both methods (XRT and CNN), the evaluation metrics, i.e. the goodness of the fit for the linear regression between ground truth characters and their predictions by XRT and CNN, are summarized in Table 1a,b (Pearson correlation coefficient (r), coefficient of determination (R^2^), mean square error (MSE), mean average error (MAE), slope and intercept). The correlations of the test set are visualized separately in Fig. 3a–d.

The test set metrics (Table 1b, Fig. 3b, d) for total kernel weight showed that both methods generated relevant predictions as both achieved R^2^ ~ 0.94 and mean absolute error (MAE) < 0.17 (MAE was slightly lower for the CNN method). The CNN model predicted the shell weight with similar accuracy (R^2^ = 0.91) as the total kernel weight (R^2^ = 0.95) but the prediction via the XRT method had notably lower R^2^ values (kernel: R^2^ = 0.93, shell: R^2^ = 0.78). Nevertheless, the MAE for the shell weight prediction was lower for XRT (MAE = 0.08) compared to CNN (MAE = 0.09). The linear regression slope on the test dataset was closer to 1 for the XRT method compared to CNN for kernel weight. The intercept was lower for CNN compared to XRT for the predictions of kernel weight and higher for CNN compared to XRT for shell weight predictions (Table 1b, Fig. 3).

**Fig. 4:**
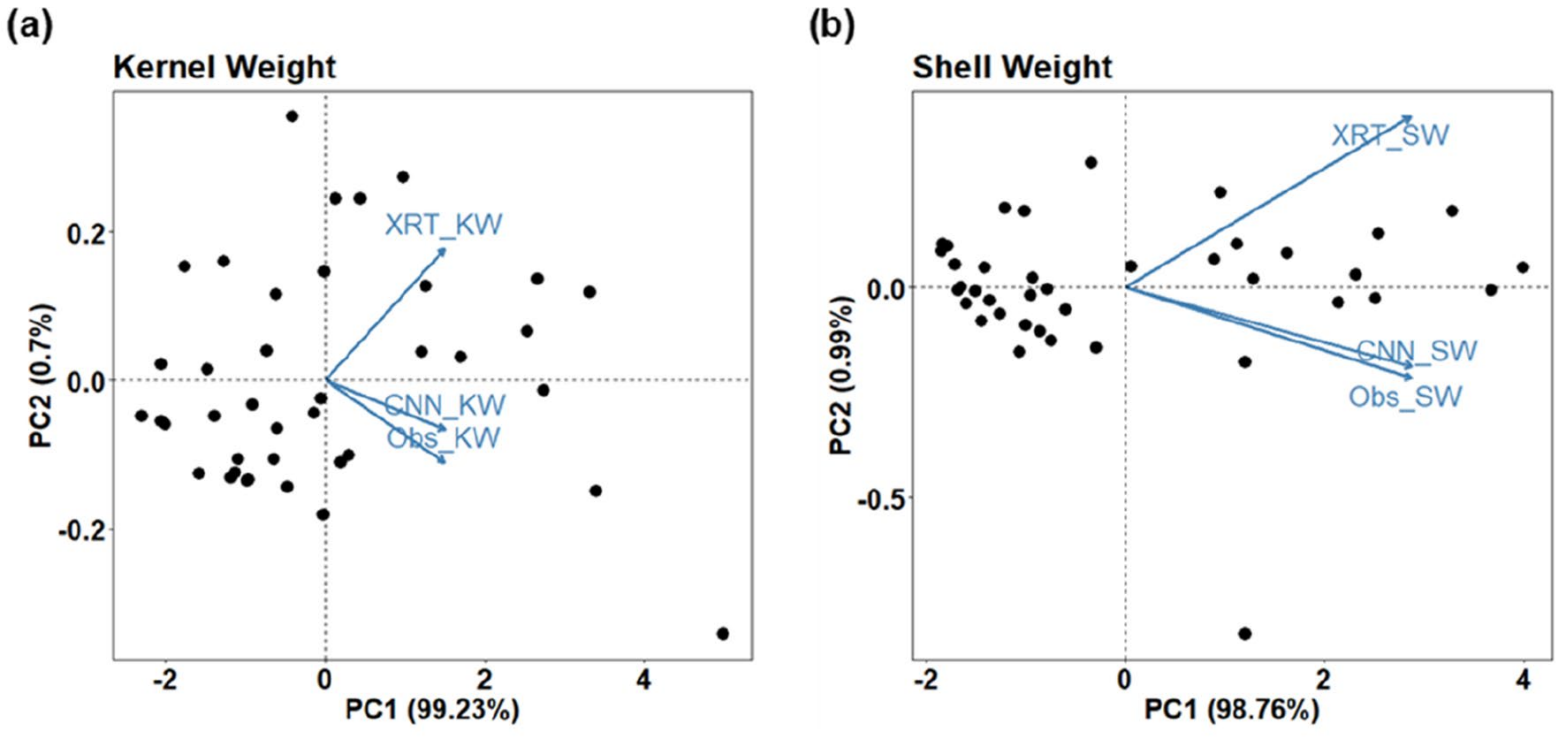
Principal Component Analysis (PCA) and visualization of two main principal components that explained > 99% of variation in kernel weight (**a**) and shell weight (**b**). The graph represents the main relations between observed kernel weight (Obs_KW) and shell weight (Obs_SW) and their prediction by XRT (XRT_KW, XRT_SW) and CNN (CNN_KW, CNN_SW)

The prediction accuracy of these two methods was also assessed using PCA biplots (Fig. 4a, b, "Feature predictions methods and comparison metrics" section). The results showed that there was generally a good agreement between predictions of features by XRT and CNN for kernel weight and shell weight (~ 99% loading on principal component 1). The principal component 2 (explaining ~ 1% variability in the dataset) pointed out that CNN predictions were closer to the ground-truth values compared to XRT for both kernel and shell weight. This method also indicated that some varieties were predicted with markedly different accuracies by XRT and CNN methods. This was apparent with genotype ICGV86564 which had the largest pods and kernels in the whole dataset (Additional file 1: Table S1).

## Discussion

### Analysis of peanut traits of economic significance

The analyzed peanut varieties encompassed a large range of variability for kernel weight (0.003–2.47 g/pod), shell weight (0.05–1.40 g/pod) and their ratio–shelling % (1.5%–87.8%), sufficiently representing the cultivated South Asian gene-pool [50, 51]. In many of the peanut market scenarios, the price incentives are primarily linked to a higher proportion of larger kernels and high shelling percentage (high proportion of kernel mass in total pod mass) in the raw produce [44, 45, 52]. Similarly, crop improvement programs have to breed for the same traits in order to enhance the economics of peanut farming. This is currently difficult because evaluation of these traits in both processes (i.e., market and breeding) relies on manual assessment which is time-consuming and potentially error-prone (e.g., [3, 44, 51,53, 54]. Therefore, assessing the potential of emerging X-ray technology options and advanced data analytics to close these gaps was the main motivation of the presented study.

### X-ray technology options for the rapid assessment of peanut commodity features

Through this study we have demonstrated that an X-ray imaging system (CTportable160.90) [43] combined with XRT- and CNN-based algorithms is a relevant technology base suitable to assist current peanut value-chains and breeding. While the use of the XRT method for similar applications in other crops has already been established [25–27, 54] its applicability for the peanut crop was tested here for the very first time. Also, the utilization of CNN algorithms for similar tasks is new and has not been attempted before.

Within our dataset, both methods (XRT and CNN) predicted the peanut produce characteristics of economic value with relevant accuracies (R^2^ > 0.94, MSE < 0.21 for kernel weight; R^2^ > 0.82, MSE < 0.1 shell weight). Although both algorithms predicted features of most of the pods similarly, the CNN predictions were closer to the ground truth observations, especially while dealing with the prediction of extreme values and contaminated pods (e.g., large pods and pods with soil remnants).

With the current system, the pod holder preparation and scanning took approximately 2 min and the prediction of features took less than 1 s (XRT) and 6 secs (CNN). Thus, a rapid estimation of the quality aspects of unshelled peanuts is possible and could support the current pod-evaluation process tremendously. However, the concrete time- and cost-efficiency of the current technology set-up were beyond the scope of this feasibility study. This will be, certainly, an important next step in the technology transfer pathway for dedicated use in the peanut value-chain and breeding. Technology interventions similar to those presented hereby could support global efforts to bridge the remaining blind spots in agricultural commodity value-chains [56]. The same technology interventions in the crop improvement process would enable faster evaluation and release of more economically beneficial cultivars.

Furthermore, the approaches currently validated on peanuts can be readily adapted to other crops where the removal of the grain shell pose difficulties for kernel features evaluations (e.g. rice, barley or oats). In such cases the "virtual shelling” enabled by X-ray technology could largely offset these hurdles. X-ray scanning is also suitable for evaluation of whole panicles (cereals) or pods (legumes) where it can offset the laborious process of grain threshing as shown before [20, 21, 25, 34, 35, 42]. Also X-ray can be used for non-destructive evaluation of inner structures and physical properties of the grains, tubers etc. Many of these are important factors related to grain processing (e.g. milling) or internal tissue health [20, 21, 27, 29, 42, 59] and automation can open new avenues to agricultural research.

## Conclusion

Many farming communities that depend on peanut production systems face numerous challenges related to irregularities in peanut produce procurement. In these market scenarios, the procurement process often begins with a slow manual shelling and weighting of peanuts at the farmers gates. Resulting delays in procurement can lead to produce contamination due to inadequate storage [47, 48]. In line with the international committee for food value and safety [3, 10, 56–58], we argue that the current procurement process could be streamlined using novel portable technologies [18, 40, 41]. Therefore, we tested the relevance of X-ray-based technology for the prediction of the key indicators of peanut produce price, kernel and shell mass. For the first time, we adapted a recently patented procedure building on a 2D-X-ray projection conversion method for biomass determination [59] and developed completely novel CNN algorithms to predict kernel and shell mass from whole peanut pod projections.

We showed both methodologies were relevant to predict the kernel and shell mass non-destructively from the 2D-X-ray pod projections. If operationalized on the ground, these could standardize and accelerate peanut commodity procurement. This acceleration would limit the risk of fungal contaminations due to inadequate storage and, at the same time, the non-destructive assessment of kernel weight, shell weight and shelling percentage would grant a fair price to farmers. The same technology could be used as a part of the peanut breeding process to accelerate the selection of economically viable products and assist peanut researchers in general. Beside the demonstrated application for the peanut procurement in India, the same technology can be adapted for assessing optically occluded features in harvested plant material. This can range from legume pods, rice grains up to the whole cereal panicles and thus offset the manual or destructive shelling or threshing process. Of course, this would need to adapt the presented imaging pipeline and algorithms used within this publication.

## Materials and methods

### Graphical overview

**Fig. 5:**
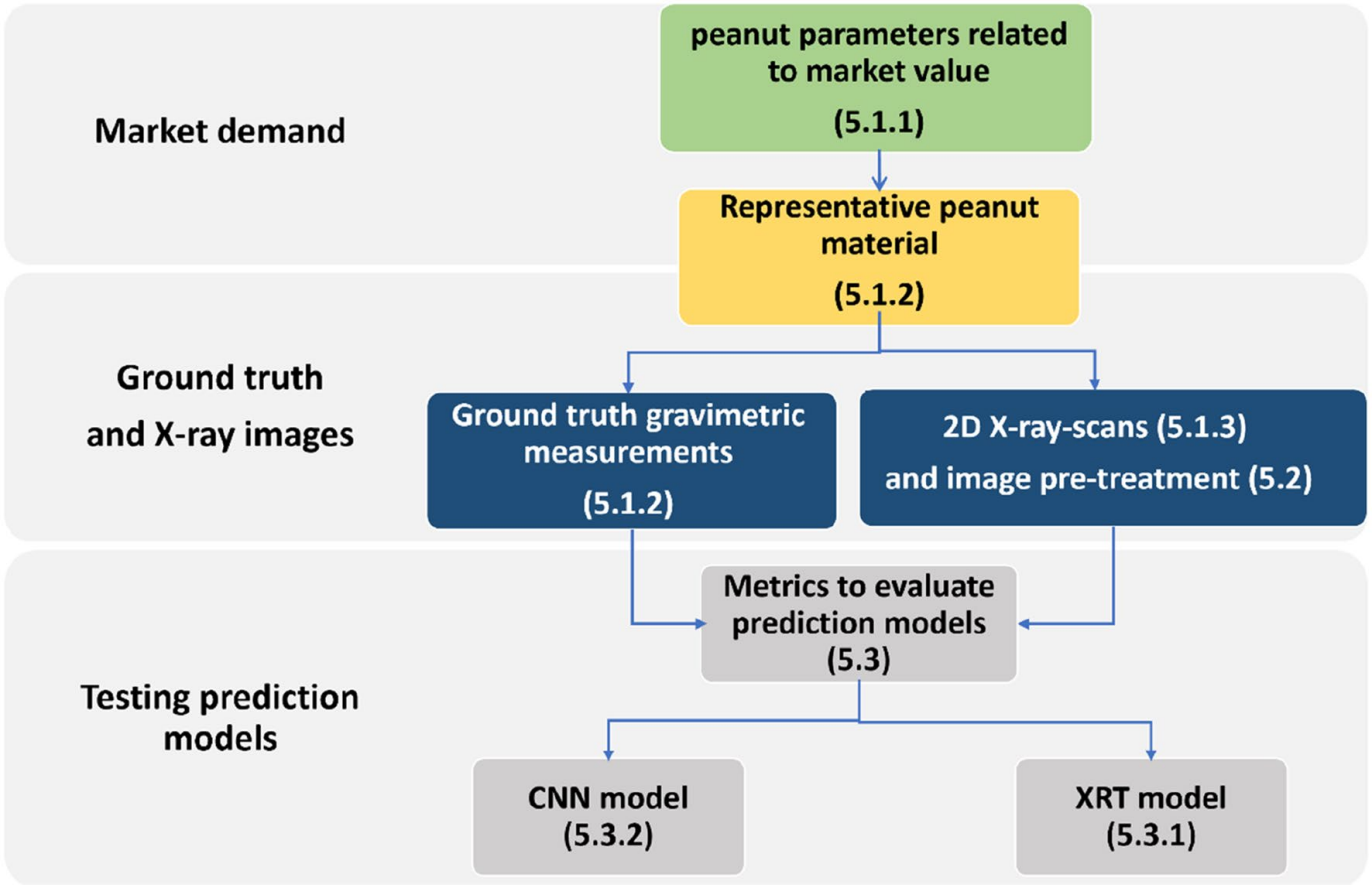
Overview about the workflow, the market demand and the derived metrics to rate the quality of peanut pods

Raw peanut procurement price typically depends on physical parameters of the peanut pods: i.e., kernel and shell mass. These are notoriously difficult to assess manually ("Peanut commodity price indicators"). We gathered peanut varieties representing the range of the peanut commodities in South Asian markets ("Peanut varieties used and ground truth measurements"). The whole pods were scanned by X-ray (2D X-ray images) and, consequently, the manual gravimetric measurements of kernel and shell mass were measured ("Peanut varieties used and ground truth measurements"). Altogether, 1752 of 2D X-ray projections of individual peanut pods were taken. The images were pre-processed ("Image treatment") and the two methods for the prediction of peanut kernel mass and shell mass trained and applied: (a) X-ray image transformation for biomass assessment (XRT method, "Features prediction using X-ray transformation (XRT) of images") and convolution neural network (CNN method, "Features prediction using CNN regression model") (Fig. 5). The standard metrics were defined to compare these two methods for their prediction accuracy, i.e., to infer peanut kernel and shell weight from pre-processed 2D X-ray scans of peanut pods ( "Feature predictions methods and comparison metrics" section).

#### Peanut commodity price indicators

The international peanut commodity market considers many traits linked to the pods and kernel features related to their physical and biochemical characters (generic guidelines for international trade, [44, 45, 58, 60]. In the selected use case for peanut value-chains (Sri Satya Sai Raithu MAC Federation Limited and commodity aggregators [61], Anantapur, Andhra Pradesh, India), only a few of these features are considered now Due to the manual processes involved in the grading, the estimation of peanut procurement cost is time consuming (typically < 30 min to assess a produce lot). Due to the lack of the storage facilities, any delay in procurement poses an additional threat to the safety of the produce (mainly the risk of fungal contaminations).

#### Peanut varieties used and ground truth measurements

To assess the technology potential, we used peanut varieties representing the range of the variability in pod and kernel sizes and shapes relevant to South Asian markets. These included 30 breeding lines and 4 released cultivars popular with Indian farmers obtained from the experimental field at the ICRISAT research station, South Asia peanuts improvement team. These have been grown under optimal irrigation and fertilization regimes in alfisol fields during the rainy season (June–September 2019). Each of the varieties contained a subset (~ 0.5 kg) of pods from several plants harvested from experimental plots. The typical trial plot size was 1.2 × 2 m.

We also included 5 peanut crops harvested from 5 different small-scale farmer fields (typically ~ 2 ha of the cropped land) in the Anantapur region in the rainy season (June–September 2019). Here, the peanut crop is typically raised on sandy soils and each farmer adapts different crop management strategies as per the available resources. We collected the sub-set of raw produce from 5 different fields (~ 0.5 kg), which contained a mix of pods from different plants. Altogether, 1752 peanut pods were analyzed. The details of these varieties and their analysis are in the Additional file 1: Table S1 and Fig. S1 a–c.

From each subset of collected peanut varieties ~ 40 pods were randomly selected. This roughly corresponds to the peanut pod quantity on which aggregators would estimate the price for the farmer's produce. Consequently, the X-ray 2D projection images of all pods were taken (details in "2D X-ray images"). Then, for each of the scanned pods, the ground truth measurements of kernel weight and shell weight were conducted gravimetrically (KERN balance, 0.001 accuracy, the evaluation by aggregators is also done gravimetrically). From the total kernel weight and the shell weight, the shelling percentage was calculated for each individual pod via $s=\frac{{W}_{k}}{{W}_{p}}\cdot 100$. In this formula, $s$ represents the shelling percentage calculated by dividing the kernel weight ${W}_{k}$ by the total weight of the pod ${W}_{p}$ and multiplying it with 100.

The distribution of all pod feature ranges (kernel weight, shell weight and shelling percentage) covered by the study were displayed in the boxplots (R software, version 4.0.2). The variation within and among the characters of different peanut varieties were visualized using the basic Quartile Box Plot method with the data distribution display (Tibco Spotfire software, version 10.7.0). The significance of differences in the peanut pod characteristics among the varieties were tested using a one-way ANOVA test followed by the Tukey–Kramer test for the pair-wise comparison of the individual varieties (Genstat software,18th Edition).

#### 2D X-ray images

2D X-ray images were taken using the CTportable160.90 system from the Development Center X-Ray Technology (EZRT) of the Fraunhofer Institute of Integrated Circuits (Fürth, Germany). The technical details of the system can be found via [25–27, 43]. In brief, the scanner consists of an X-ray source with acceleration voltages ranging from 30 to 90 kV, a current up to 160 µA and a detector size of 2304 × 1300 pixels (49.5 μm pixel size). The sample stage can be positioned between the X-ray source and the detector with a minimum focus object distance (FOD) of 16 mm and a maximum FOD of 285 mm, resulting in a maximum resolution of about 2.8 μm. The detector is a 14-bit CMOS sensor (Teledyne DALSA Shad-o-Box 3 K HS) featuring a direct-contact Gd2O2S scintillator (Kodak Min-R 2190) scintillation foil.

To test the minimum technology requirements for feature extractions, only 2D projections of peanut pods were used. For this, the peanut pod holder was designed and crafted from extruded polystyrene (eps) to hold 4 peanut pods at the time. To cover the largest field of view, the pod holder was fixed directly onto the detector, resulting in an optical magnification close to 1. The system was operated at 60 kV and 103 µA with an exposure time of 300 ms resulting in a resolution of about 49.5 µm. A total of 438 2D X-ray projections were taken with 4 pods in the holder resulting in 1752 individual pods. Including changing the pod holder, system preparation and measurement, each scan took about 2 min. The system functionalities were controlled by the software Volex10 (Fraunhofer Institute of Integrated Circuits, Germany [62]).

### Image treatment

We took altogether 438 projections of the eps grid organized to carry four separate pods (1752 pods). Each image was cropped into four to display just one peanut pod and exported into an image format (TIFF). The raw images consisted of gray pixel values in a range from 5597 to 707 (mean of pixel values 4906.43).

**Fig. 6:**
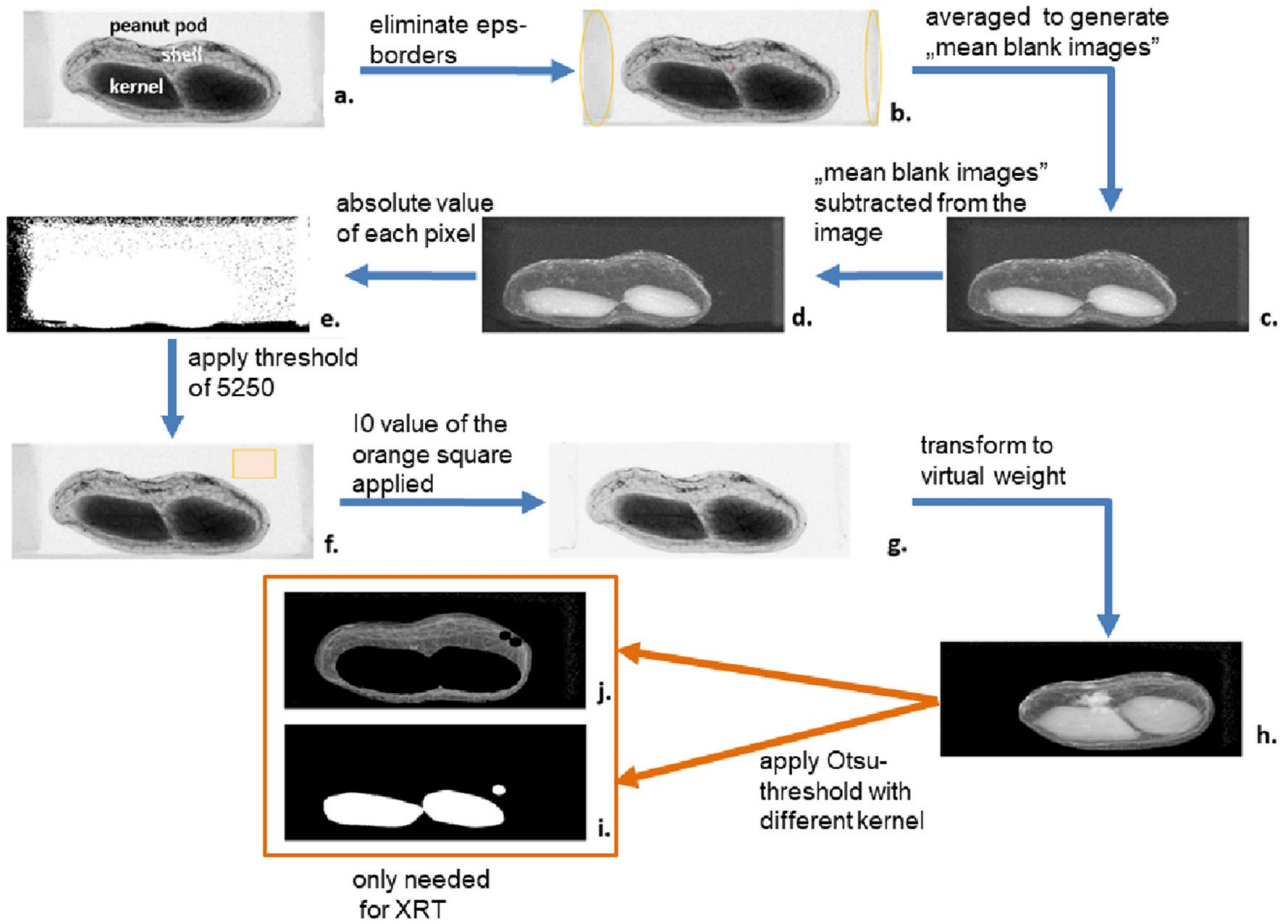
Individual steps involved in image processing of the individual X-ray pod projections obtained from CTportable160.90. **a** is the original image (within the image the parts of the pods that we attempted to predict are named). In the following steps the eps-borders were eliminated; these borders are marked with yellow ellipsoids (**b**); subsequently, the areas of borders not containing a peanut pod were averaged (**c**) and subtracted from the fig (**d**) (compare **c** and **d**). Consequently, for each pixel its absolute value was taken (**e**) and a threshold value of 5250 (**c**) applied to the image leading to (**f**). In **f** the small area not containing peanut or a border (indicated by an orange square) was used to set the i0. This i0 was applied to the fig and resulted in **g**. Consequently, the image was transformed with Eq. 1 used as an input to CNN (**h**). To apply the XTR method for pod feature prediction, the mask based on the Otsu-thresholding method—used to differentiate the kernel (**i**) and shell occupied area (**j**)—was further applied to 5h

The following steps were designed to create labels and compensate for the absorbance of the residual eps-grid projections (i.e., “border”; Fig. 6b). For this, two projections containing the empty eps grid were taken and exported as a TIFF image. Images were averaged to generate “mean blank images" (see Fig. 6c). The mean blank images were then subtracted from all the peanut images. After the subtraction of the mean blank images, some pixels had negative values. To eliminate them, each pixel of each image was squared, and the square root was taken (Fig. 6d). Afterwards, to create labels of the residuals of the eps-grid, a threshold of 5250 was iteratively determined within a subset of randomly chosen peanut images which were visually checked. All values below this threshold were set to 0 (black), the rest to a value of 65,535 (white/ highest unsigned integer value, Fig. 6e).

For each image, the mean gray value of a small area (~ 40 × 30 pix) outside of the projected peanut was calculated (named ${i}_{0}$; Fig. 5f). The highest ${i}_{0}$ value was 5382.635, the lowest 4864.444, (these values actually represent the stability of the X-ray source operated with the settings as described above).

Consequently, the image areas containing the residual eps grid were set to the ${i}_{0}$ value (Fig. 6g). Thus, the following steps were not affected heavily from the sample holder. However, as the threshold for the label was selected manually, some of the images contained some artefacts. For the XRT and CNN method, the raw grey values within a pixel were transformed into a virtual weight estimation Fig. 6h [25].

Using this approach, the exponential absorption of X-rays within matter is corrected and transformed into a linear space correlating with the actual biomass of the absorbing matter. In this case, correlating with the total biomass of shell and peanut kernels. All values below 0 were corrected to 0 taking the intrinsic noise within the X-ray projections into account. After implementing this routine, all grey values ranged between 0 and 2. These images (as in Fig. 6h) served as direct input training data for the CNN (see Sect. "Features prediction using CNN regression model").

Further image processing steps were specifically done to extract the peanut pod features via the XRT method ("Features prediction using X-ray transformation (XRT) of images"). This required the separation of the image area occupied by the kernel and the shell. For this, a threshold was set individually for every preprocessed image (Fig. 6h) using the automatic threshold algorithm by Otsu [62]. The Otsu algorithm exhaustively searches for the threshold that maximizes the inter-class variance. In our case, the application of the Otsu thresholding method (Eq. 1) found the threshold distinguishing the image background from the kernel [63]. This algorithm found the applicable threshold minimizing the following equation.

1 $${{\sigma }^{2}}_{w}(t)={w}_{0}(t){{\sigma }^{2}}_{0}(t)+{w}_{1}(t){{\sigma }^{2}}_{1}(t)$$

As we aimed to define the kernels it was necessary to choose the threshold in such a way that the shell would become part of the background. After the iterations of the Otsu thresholding method, the shell was still part of the foreground. As we only wanted the kernels to be in the foreground, and the shell appeared slightly lighter, the value 50 was found suitable and finally subtracted from the Otsu threshold. This value 50 was obtained manually using a small set of peanut images to shift the threshold so that the pixel representing the shell became part of the background. All values above the threshold were set to 0 (black), all values below to 1 (white). On the resulting binary images an ellipse shaped filter mask shape = (5 × 5 pixel) was used for a morphological erosion. Additionally, the morphological dilation was performed as well, with an ellipse-shaped filter mask (shape: 9 × 9 pixel) [64] (see Fig. 6i).

The resulting binary peanut kernel images (Fig. 6i) were used to label the peanut kernels and the peanut shells of the transformed images (Fig. 6h), respectively. In the process, the binary images (Fig. 6i) were multiplied with the transformed images (Fig. 6h) to obtain only the pixels representing the kernel. The results were integrated and plotted against the actual kernel weight. For the shell weight, the binary peanut images (Fig. 6i) were used inverted to serve as a label image for the shell (Fig. 6h). All bright pixels in the label image denote foreground objects whereas dark pixels denote the image background (compare Fig. 6j). The resulting images were also integrated and plotted against the actual shell weight.

### Feature predictions methods and comparison metrics

After the pretreatment of the images the dataset was randomly separated into a calibration and testing dataset 90:10%. The “calibration” dataset (90% of the data) was used to train the feature predictive algorithms and the “testing” dataset (10% of the data) to generate the metrics that indicate the predicting power of each method. For each trait (kernel weight, shell weight) and method, descriptive statistics of the relationship between ground truth measurements and predicted values were calculated, i.e., r (Pearson’s correlation coefficient), R^2^ (coefficient of determination), MSE (mean squared error), MAE (mean absolute error), slope, intercept of relation between the ground-truth observations and predictions. The exact same pod images were used as a “calibration” and “test” set to compare the prediction power of the two tested methodologies ("Features prediction using X-ray transformation (XRT) of images" and "Features prediction using CNN regression model").

To compare the features extraction methodologies further, principal component analysis (PCA) between the ground-truth measurements and the features predicted using methods "Features prediction using X-ray transformation (XRT) of images" and "Features prediction using CNN regression model" was performed using R Studio software (v2021.09.0 Build 351). PCA helped to clarify the source of prediction errors for particular types of peanut pods and pointed out further prediction methodology improvements, limitations and advantages of each method.

#### Features prediction using X-ray transformation (XRT) of images

To separate the kernel and shell biomass, the label images were applied on the processed images to dissect the pixels reflecting the peanut kernels and shells, respectively (details in "Image treatment" “label image” application process is visualized on Fig. 6h–j.). For all images in the calibration set (90% of dataset containing the same pod images as the calibration set used for CNN, "Features prediction using CNN regression model"), the grey pixel values belonging to kernel and shell projections were integrated and correlated and the actual peanut kernel and shell weight measured gravimetrically. The reliability of this prediction method was expressed through linear regression parameters between XRT predictions and gravimetric measurements for calibration (90%) and test portions (10%) of the dataset. These metrics from the linear regression are summarized in the Additional file 1: Table S1 and displayed in Fig. 3.

#### Features prediction using CNN regression model

The calibration dataset (i.e., 90% of the complete data set) was further split into a “training” (80% of calibration set) and “validation” set (20% of the calibration set, which was used to monitor network accuracy during the training on 80% of the calibration dataset after each epoch).

CNNs are a special kind of deep neural network designed to identify features in 2D images where numerous different mask filters are trained to identify recurring structures. To train a network, a training set is needed that contains the input images and the associated target values (i.e., ground truth measurements; see "Peanut varieties used and ground truth measurements"). Several input images (“training set”, Fig. 6h., "Image treatment" section) are fed into a pre-specified neural network structure. In the last layer, one or more output values are calculated. The output is then compared to the target values. The resulting error is backpropagated through the network to optimize the different parameters in the model structure [65].

In our case we constructed the CNN network training structure to predict the peanut kernel and shell weights from virtual biomass images (Fig. 6h). This specific network consisted of 10 convolutional layers, maximum pooling layers and fully connected layers/dense layers. The output layer consisted of two output neurons, featuring the predicted kernel and shell weight. In contrast to classic feed forward CNNs the output in this study consisted of continuous values. Hence, we refer to our network as a CNN regression model.

For this study a structure similar to the AlexNet was built [66–69]. The first convolutional layer filtered the input image with 96 filter masks (size: 11 × 11 pixels) with a stride of 4 × 4. The second convolutional layer took the max pooled output of the first convolutional layer as an input and filtered it with 256 filter masks (size: 5 × 5 pixels). The third, fourth, and fifth convolutional layers were connected without any intervening pooling layers. The third convolutional layer had 384 filter masks (size: 3 × 3 pixels) connected to the pooled outputs of the second convolutional layer. The fourth convolutional layer also had 384 filter masks of 3 × 3 pixels in size, and the fifth convolutional layer had 256 filter masks (size: 3 × 3 pixels). All fully connected layers had 200 neurons each. The first two fully connected layers also had a dropout of 50%. The last output layer consisted of two output neurons each trained to predict the peanut kernel and shell weight. The two maximum pooling layers between the first three layers had a size of 3 × 3 pixels and a stride of 2 × 2 [70]. All layers had “valid padding” meaning no padding and were activated with the ReLu activation function [71]. The loss function used was the MSE (mean squared error). During training, the MAE (mean average error) between target and predicted values was also monitored at the end of each training step. The batch size used for training was 50 images fed into the network during one training step. The optimizer of choice for updating weights (filter masks) and biases was Adaptive Moment estimation (ADAM), with a leaning rate of 0.00005, the exponential decay rates ${\beta }_{1}$ = 0.9 and ${\beta }_{2}$ = 0.999 and a convergence criterion $\epsilon =1\cdot {10}^{-8}$ [72].

In our case, CNN was trained for 30 epochs with virtual biomass images and their corresponding peanut kernel and shell weight values as target values. Each value of the two measured parameters—shell and kernel weight values—was assigned a “sample weighting “. This value was implemented to account for peanut kernel and shell weights that are underrepresented in the training set (refer to "Peanut varieties evaluation for price indicators: kernel weight, shell weight, shelling percentage" section). It is defined as the percentage of how much the particular loss was weighted during the back-propagation process. The sample weighting was adjusted according to the number of occurrences of a specific peanut kernel and shell weight in the overall distribution. Thus, extreme values of weights for kernel and shell—that were underrepresented in the dataset—were assigned a higher sample weight than more abundant examples. This way of sample weighting assigns a higher loss to underrepresented ranges of values increasing their impact on the learning step. Before the training was initiated, the weights (filter masks) and biases of the convolutional and fully connected layers were initiated. The biases were initiated with zeros. The weights were initialized with the Glorot uniform initializer, also called Xavier uniform initializer [73]. The hardware used was the GPU GeForce GTX 1050 Ti and 16 GB RAM.

## Supplementary Information

**Additional file 1:** **Figure S1 a, b, c:** Modified boxplot showing variation in kernel weight **(a)**, shell weight **(b)**, and shelling percentage **(c)** within and across 39 peanut genetic materials used in the study: advanced breeding lines (grey), elite cultivars (blue) and farmer-produced peanut crop (green). Each boxplot represents one particular genetic material. Within each boxplot the mean of each genetic material is marked by red line (−) and the values distribution within particular genetic material is shown along the vertical axes of the boxplot. Dashed line (---) depicts the average of all 39 genetic materials used in this study. **Table S1: **The table contains the list of peanut genetic materials used for in this study. These include peanut crop harvested from farmers in Anantapur (sequential number 1-5), elite cultivars formally released and currently cultivated across India (sequential number 6-9), advanced breeding lines obtained from the ICRISAT peanut breeding team (sequential number 10-39). Each of the genetic materials consisted of ~40 peanut pods which were gravimetrically evaluated for kernel weight, shell weight and shelling percentage. The means of these pod characters for each genetic material are presented in the table along with the results of the Tukey-Kramer test (i.e. the letters accompanying the means). The same letters occurring in the letter sequence indicate that the pod characteristics of the genetic material were not significantly different and vice versa.

## Data Availability

The ground-truth dataset(s) supporting the conclusions of this article will be made available in the data repository after the peer-review process; www.icrisat.dataverse.org. The peanut varieties used in this study can be made available upon request after adhering to the international standards for genetic material transfer.

## Abbreviations

ANOVA: Analysis of variance
CNN: Convolutional neural network
CT: Computed tomography
MAE: Mean absolute error
MSE: Mean squared error
XRT: External radition therapy
