# Supplementary material for: X-ray driven peanut trait estimation: computer vision aided agri-system transformation
Source: Plant Methods. 2022 Jun 6;18:76. doi: 10.1186/s13007-022-00909-8 (PMC9169268; doi:10.1186/s13007-022-00909-8)
Supplement: Supplementary file 1 — Additional file 1: Figure S1 a, b, c: Modified boxplot showing variation in kernel weight (a), shell weight (b), and shelling percentage (c) within and across 39 peanut genetic materials used in the study: advanced breeding lines (grey), elite cultivars (blue) and farmer-produced peanut crop (green). Each boxplot represents one particular genetic material. Within each boxplot the mean of each genetic material is marked by red line (−) and the values distribution within particular genetic material is shown along the vertical axes of the boxplot. Dashed line (---) depicts the average of all 39 genetic materials used in this study. Table S1: The table contains the list of peanut genetic materials used for in this study. These include peanut crop harvested from farmers in Anantapur (sequential number 1-5), elite cultivars formally released and currently cultivated across India (sequential number 6-9), advanced breeding lines obtained from the ICRISAT peanut breeding team (sequential number 10-39). Each of the genetic materials consisted of ~40 peanut pods which were gravimetrically evaluated for kernel weight, shell weight and shelling percentage. The means of these pod characters for each genetic material are presented in the table along with the results of the Tukey-Kramer test (i.e. the letters accompanying the means). The same letters occurring in the letter sequence indicate that the pod characteristics of the genetic material were not significantly different and vice versa. [file 13007_2022_909_MOESM1_ESM.docx]

**Additional file 1: Figure S1 a, b, c:** Modified boxplot showing variation in kernel weight **(a)**, shell weight **(b)**, and shelling percentage **(c)** within and across 39 peanut genetic materials used in the study: advanced breeding lines (grey), elite cultivars (blue) and farmer-produced peanut crop (green). Each boxplot represents one particular genetic material. Within each boxplot the mean of each genetic material is marked by red line (−) and the values distribution within particular genetic material is shown along the vertical axes of the boxplot. Dashed line (---) depicts the average of all 39 genetic materials used in this study.

**Additional file 1: Table S1:** The table contains the list of peanut genetic materials used for in this study. These include peanut crop harvested from farmers in Anantapur (sequential number 1-5), elite cultivars formally released and currently cultivated across India (sequential number 6-9), advanced breeding lines obtained from the ICRISAT peanut breeding team (sequential number 10-39). Each of the genetic materials consisted of ~40 peanut pods which were gravimetrically evaluated for kernel weight, shell weight and shelling percentage. The means of these pod characters for each genetic material are presented in the table along with the results of the Tukey-Kramer test (i.e. the letters accompanying the means). The same letters occurring in the letter sequence indicate that the pod characteristics of the genetic material were not significantly different and vice versa.

| S no | Type | Genetic Material | Kernel weight (g) | | Shell weight (g) | | Shelling percentage (%) | |
| --- | --- | --- | --- | --- | --- | --- | --- | --- |
| 1 | **Farmer_produce** | Anantapur_F_mallesh | 0.78 | bcdefg | 0.26 | bcdefgh | 71 | efgijklmno |
| 2 |  | Anantapur_F_Naveen_K6 | 0.62 | abcde | 0.19 | abcdef | 76 | no |
| 3 |  | Anantapur_F_Shilpa | 0.68 | abcde | 0.27 | cdefgh | 70 | defghijklmn |
| 4 |  | Anantapur_FPO_Natraj | 0.64 | abcde | 0.22 | abcdef | 73 | jklmno |
| 5 |  | Anantapur_FPO_pooled pods | 0.80 | cdefg | 0.36 | hi | 68 | bdefghijklmn |
| 6 | **Elite cultivar** | CSM-1 | 0.44 | a | 0.16 | abcd | 70 | defghijklmn |
| 7 |  | GG20 | 0.66 | abcde | 0.16 | abc | 79 | o |
| 8 |  | GJG- HPS-1 | 0.85 | defgh | 0.29 | fgh | 71 | fgijklmno |
| 9 |  | Sunoleic 95-R | 0.74 | abcdefg | 0.22 | abcdef | 74 | lmno |
| 10 | **Advanced breeding line** | ICGV05100 | 0.64 | abcde | 0.19 | abcdef | 73 | jklmno |
| 11 |  | ICGV171007 | 0.69 | abcde | 0.35 | ghi | 63 | abcdef |
| 12 |  | ICGV171017 | 0.87 | defgh | 0.29 | fgh | 73 | jklmno |
| 13 |  | ICGV171023 | 0.72 | abcdef | 0.28 | efgh | 67 | bcdefghijklmn |
| 14 |  | ICGV171303 | 1.39 | lm | 0.74 | oq | 65 | abcdefghijk |
| 15 |  | ICGV171345 | 1.30 | kl | 0.76 | q | 62 | abcd |
| 16 |  | ICGV171395 | 1.21 | ijkl | 0.70 | nopq | 63 | abcdef |
| 17 |  | ICGV171396 | 0.99 | fghij | 0.51 | jkl | 64 | abcdefghi |
| 18 |  | ICGV171413 | 1.25 | jkl | 0.63 | mno | 66 | abcdefghijkl |
| 19 |  | ICGV181006 | 0.47 | ab | 0.19 | abcdef | 69 | defghijklmn |
| 20 |  | ICGV181014 | 0.57 | abcd | 0.28 | efgh | 63 | abcdefg |
| 21 |  | ICGV181015 | 0.67 | abcde | 0.19 | abcdef | 75 | no |
| 22 |  | ICGV181032 | 0.49 | ab | 0.23 | abcdef | 65 | abcdefghijk |
| 23 |  | ICGV181033 | 0.48 | ab | 0.14 | a | 72 | gijklmno |
| 24 |  | ICGV181035 | 0.58 | abcd | 0.17 | abcde | 73 | jklmno |
| 25 |  | ICGV181036 | 0.49 | abc | 0.14 | ab | 74 | lmno |
| 26 |  | ICGV181085 | 0.56 | abcd | 0.22 | abcdef | 70 | defghijklmn |
| 27 |  | ICGV181088 | 0.82 | defgh | 0.27 | defgh | 74 | klmno |
| 28 |  | ICGV181090 | 0.67 | abcde | 0.28 | efgh | 70 | defghijklmn |
| 29 |  | ICGV181119 | 0.59 | abcd | 0.24 | abcdefg | 69 | bdefghijklmn |
| 30 |  | ICGV181133 | 0.71 | abcdef | 0.26 | cdefgh | 71 | fgijklmno |
| 31 |  | ICGV181269 | 0.78 | bcdefg | 0.45 | ij | 60 | ab |
| 32 |  | ICGV181275 | 0.69 | abcde | 0.46 | ij | 58 | a |
| 33 |  | ICGV181282 | 0.93 | efghi | 0.51 | jkl | 62 | abcde |
| 34 |  | ICGV181285 | 1.07 | hijk | 0.57 | klm | 63 | abcdefgh |
| 35 |  | ICGV181294 | 0.78 | bcdefg | 0.43 | ij | 64 | abcdefghij |
| 36 |  | ICGV181321 | 1.21 | ijkl | 0.62 | lmn | 66 | abcdefghijklm |
| 37 |  | ICGV181322 | 0.85 | defgh | 0.49 | jk | 61 | abcd |
| 38 |  | ICGV181326 | 1.00 | ghij | 0.64 | mnop | 60 | abc |
| 39 |  | ICGV86564 | 1.63 | m | 0.54 | jklm | 75 | lno |
